# Quantifying the Variability of Ground Light Sources and Their Relationships with Spaceborne Observations of Night Lights Using Multidirectional and Multispectral Measurements

**DOI:** 10.3390/s23198237

**Published:** 2023-10-03

**Authors:** Noam Levin

**Affiliations:** 1 Department of Geography, Hebrew University of Jerusalem, Jerusalem 91905, Israel; noamlevin@mail.huji.ac.il; 2 Remote Sensing Research Center, School of the Environment, University of Queensland, St Lucia, QLD 4072, Australia

**Keywords:** night lights, multispectral, multidirectional, SDGSAT-1, LANcube

## Abstract

With the transition to LED lighting technology, multispectral night-time sensors are needed to quantify the changing nightscapes, given the limitations of the panchromatic sensors. Our objective was to quantify the contribution of lighting sources as measured on the ground and examine their correspondence with night-time brightness and color as measured from space. We conducted ground-based measurements of night-time brightness using the multidirectional (top, rear, right, front, left) and multispectral LANcube v2, which was mounted on the roof of a car, over 458 km of roads in central Israel and in Brisbane, Australia. For spaceborne measurements, we used the SDGSAT-1 multispectral Glimmer sensor. We found that spaceborne measurements of apparent radiance were best explained when including all ground-based directional measurements, with greater explanatory power for highways (R^2^ = 0.725) than for urban roads (R^2^ = 0.556). Incoming light in the five directions varied between road classes and land use. In most cases, the variability in night-time brightness and color was greater for urban road sections than for highways. We conclude that due to the spectral mixture of lighting sources, at a medium spatial resolution, the impact of the transition to LED lighting may be more easily recognized from space over highways than in dense urban settings.

## 1. Introduction

Quantifying human activity from space and understanding its spatial and temporal patterns is one of the main challenges we are facing as the world’s population is becoming more urban [1]. Remote sensing of night lights is now recognized as one of the key approaches for observing anthropogenic activity, and the number of available satellites which enable us to measure night-time lights is growing constantly, along with their uses [2,3]. In this study, our objective was to quantify the contribution of lighting sources as measured on the ground and examine their correspondence with night-time brightness and color as measured from space.

With the transition of lighting technologies to LED (light-emitting diodes), the limitations of panchromatic night-time sensors, such as DMSP/OLS (Defense Meteorological Satellite Program/Operational Line Scanner) and VIIRS/DNB (Visible Infrared Imaging Radiometer Suite/Day–Night Band) are becoming more evident, given their inability to discern between lighting types and given that they do not cover the blue band, in which many LED lights have an emission peak [4]. Until recent years, there was only one source of multispectral remote sensing of night lights from space, that of astronaut photographs from the International Space Station [5]; however, their acquisition is not systematic and few areas are covered by them [2]. Currently there are two Chinese satellite missions which provide multispectral remote sensing of night lights, the commercial Jilin-1 (at a spatial resolution of ~1 m; [6]), and the SDGSAT-1 (Sustainable Development Goals Satellite, at a spatial resolution of 40 m; [7,8]), enabling us to study sources of artificial lights in fine detail.

The factors explaining night-time light variability range from factors at the country level (e.g., gross domestic product; [9,10]), socio-economic variables at the locality level [11], and land use, building heights, and specific lightings installed at the suburb and street level [12,13]. Given the variation in satellites’ viewing angles within and between images and the anisotropic nature of light emissions, which affects night-time brightness as measured from above [14,15], multiple angle observations are very valuable to better understand land use patterns based on night-time lights and for the study of light pollution [16]. Ground-based measurements are highly important to better understand and quantify the exposure of people and other living organisms to artificial light at night, given that satellite based night-time imagery mostly captures light which is emitted upwards and not light which is emitted horizontally [17]. Quantifying the light which is emitted both horizontally and vertically is important to understand the sources of light emission and their relative contribution in different settings (e.g., urban vs. rural, commercial vs. residential) and to better understand the factors explaining night-time lights as observed from space. For example, estimations of the contribution of street lighting to the overall emissions of lights from cities upwards were found to vary between 12% and 100%, depending on the city which was studied and the methodology used for estimating this [18]. To measure both vertical and horizontal light emissions, ground observations are essential [19], and various types of ground devices were used and developed for this, including DSLR (digital single-lens reflex) cameras with a fisheye lens [20] and sky quality meters (SQM; [21]).

To better understand the lighting sources contributing to night light as measured from space, we combined ground measurements along selected routes that we collected using the recently developed photometer known as the LANcube v. 2 [22], together with night-time images acquired by the SDGSAT-1.

More specifically, our objectives were the following:

- To examine the differences in night-time lights as a function of road type and of urban land use.
- To examine the differences in the magnitude and color of night-time lights as measured in different directions, comparing roads in urban vs. rural areas.
- To examine whether measurements of night-time brightness in the horizontal direction improve our ability to explain night-time lights as measured from space.

We hypothesized that:

- Highways belonging to a higher hierarchy would be more brightly lit than other road types and residential streets.
- Spaceborne measurements of night lights would be better explained by including ground based horizontal light emissions.
- The correlations between night-time brightness and color as measured in different directions will be low.
- The relative contribution of light from the different directions will vary as a function of the class of the road.
- In commercial areas, the contribution of horizontal light (as measured on the ground, especially to the left and right) to measured brightness will be greater than in residential areas.
- It will be easier to detect the type of street lighting from space outside of urban areas, given the multiple sources of artificial lights in urban areas. Therefore, we hypothesized that the variability in lighting, both in terms of its brightness and color using the metrics of the MSI (melatonin suppression index) and R/G ratio (red/green ratio) will be greater in urban road sections than in non-urban road sections.

## 2. Materials and Methods

### 2.1. Study Area

**Figure 1:**
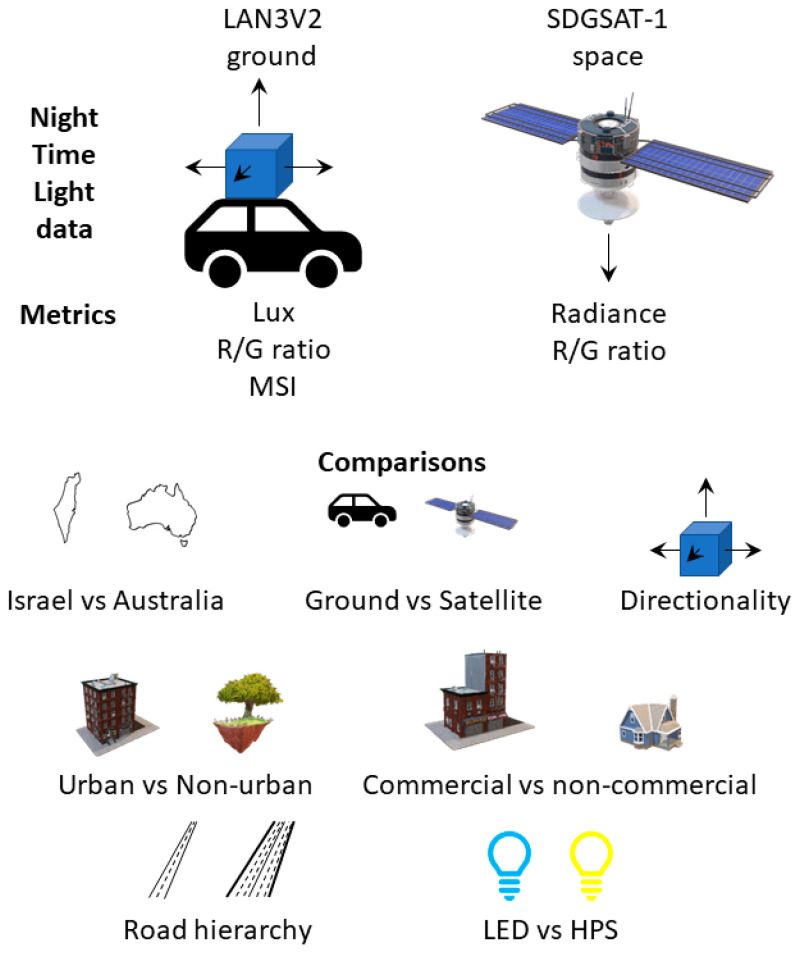
Schematic flowchart of the night-time light data sources used in this study, the metrics derived from them, and the comparisons which we conducted based on these metrics. R/G ratio stands for red/green ratio, and MSI stands for the melatonin suppression index.

**Figure 2:**
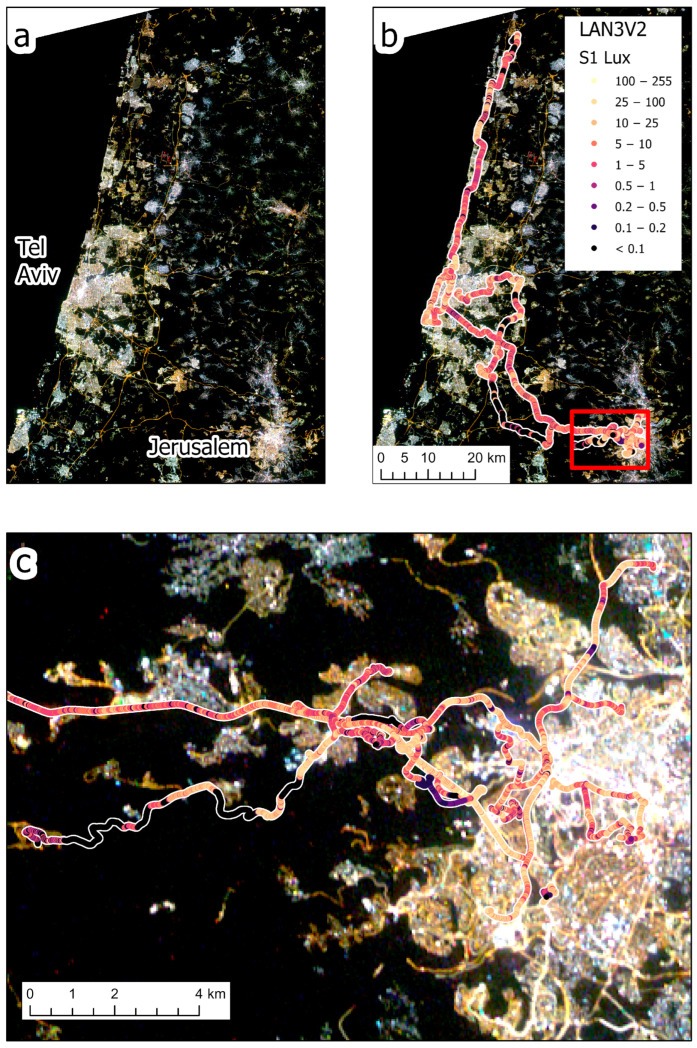
The layout of the 11 measurement routes in central Israel, colored by the lux values of the top sensor (S1) of the LAN3V2 which was facing upwards. In the background is a night-time image of SDGSAT-1, acquired on 27 April 2023. (**a**) shows the SDGSAT-1 on its own, whereas in (**b**) the satellite image is overlaid by the lux measurements of the S1 sensor. (**c**) provides a zoom-in of Figure 1b in the area of western Jerusalem and its suburbs.

**Figure 3:**
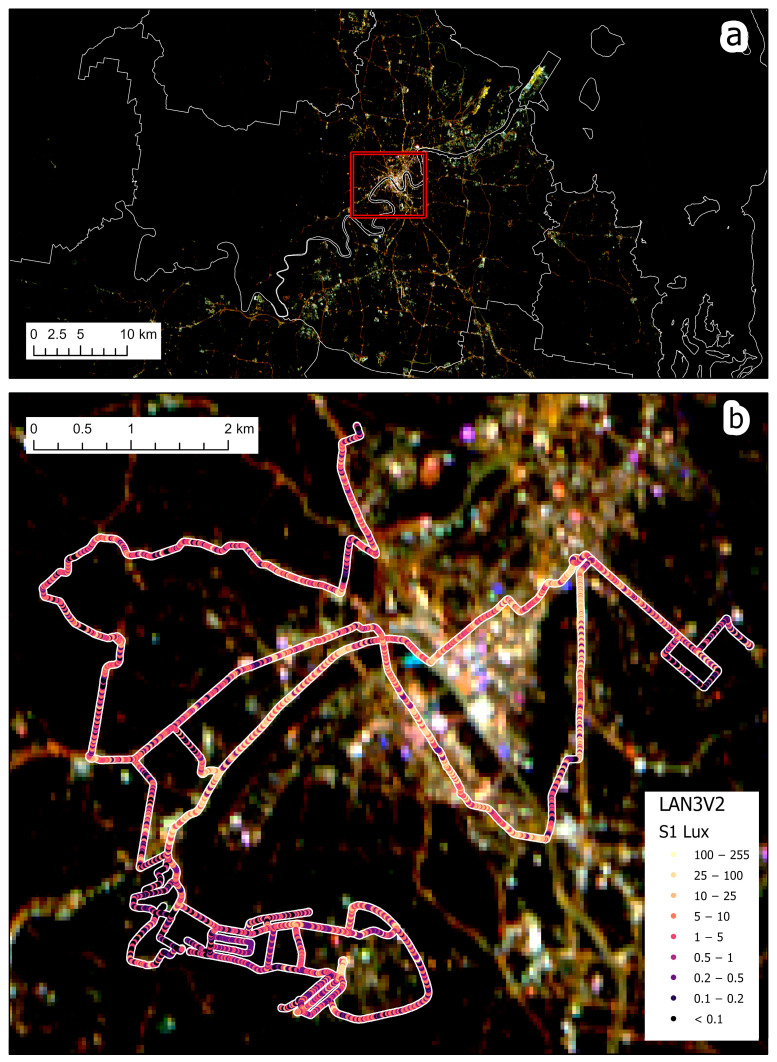
The layout of the 4 measurement routes in Brisbane, Australia, colored by the lux values of the top sensor (S1) of the LAN3V2 which was facing upwards. In the background is a night-time image of SDGSAT-1, acquired on 18 July 2022. (**a**) shows the SDGSAT-1 on its own, whereas in (**b**) the satellite image is overlaid by the lux measurements of the S1 sensor.

In this study we included night-time measurements from central Israel and from Brisbane, Australia, allowing us to also compare a country whose cities are brightly lit (Israel) and a country in which streets are lit at relatively lower levels (Australia) [10] (Figure 1). Our measurement routes in Israel included both urban streets and highways (both lit and unlit) (Figure 2), whereas our measurement routes in Australia were all within the city of Brisbane (Figure 3). Both countries are now transitioning street lights to LED technology, from high pressure sodium (HPS) lights in Israel and from HPS, compact fluorescent lamps (CFL), mercury, metal–halide, and other lighting technologies in Brisbane, Australia.

### 2.2. Data Sets

#### 2.2.1. Ground Measurements

**Figure 4:**
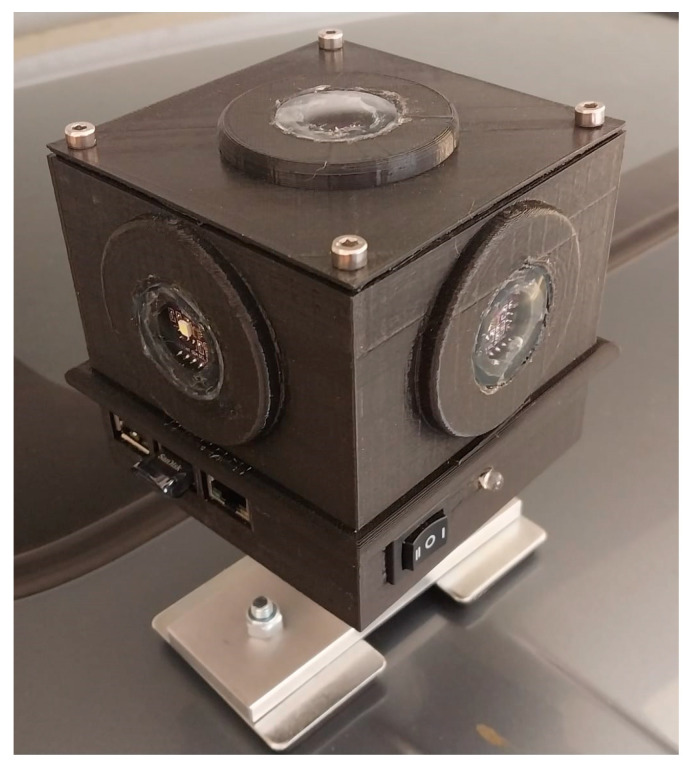
The LANcube v2 (LAN3V2) as mounted on the roof a car.

We measured night-time brightness using the LANcube v2 (LAN3V2)—a device developed by [22], which allows sampling and measuring multispectral (red, green, blue and clear—i.e., the entire visible range) and multidirectional direct artificial light (Figure 1). The multidirectional sensors of the LAN3V2 are named as follows: S1 (top), S2 (rear), S4 (front), S3 (right), S5 (left) (Figure 4). The LAN3V2 was mounted on the roof of a car and was connected to a GPS, allowing the measurement of the coordinates of all light samples, which were acquired every 0.6 s. The field of view of the LAN3V2 is very close to a cosine function hence, it is very wide. The minimum light level detected by the LAN3V2 is estimated to be at the order of 0.015 lux (signal to noise ratio ≥ 3) [22]. The LAN3V2 is calibrated and provides for each direction the following variables for each measurement: *gain*, *acquisition time* (ms), *color-correlated temperature* (*CCT*; K), melatonin suppression index (*MSI*), *lux*, *red*, *green*, *blue*, *clear*, and *flag* (OK, UnderExposed, OverExposed, ERror). The raw color signals are in digital numbers but can be compared after calibrating them by dividing the DN (digital number) values by the gain and by the integration time (the calibrated bands are hence named *Rcal*, *Gcal*, and *Bcal*).

The lux is calculated by the LAN3V2 using the following formula (Martin Aubé, personal communication):

*L* = (−0.32466 × *red*) + (1.57837 × *green*) + (−0.73191 × *blue*) (1)

*Lux* = *L/gain/acquisition_time* × 395 (2)

In addition to the variables which were provided and calculated automatically by the LAN3V2, we calculated the following variable, for each measurement for each of the sensors:

*R*/*G* *ratio* = *Rcal*/*Gcal* (3)

To evaluate the overall artificial light to which the LAN3V2 was exposed, and its sources, we calculated the following variables:

*Lux sum* = sum of *lux* values in all five directions for a single measurement (4)

*Lux S1 pct* = *Lux of S1* [top]/*Lux sum* (5)

*Lux S1 S3S5 ratio* = *Lux of S1* [top]/(*Lux of S3* [right] + *Lux of S5* [left]) (6)

*Lux S2S4 S3S5 ratio* = (*Lux of S2* [rear] + *Lux of S4* [front])/ (*Lux of S3* [right] + *Lux of S5* [left]) (7)

We removed from the analysis all erroneous measurements (e.g., measurements for which GPS coordinates were missing or where erroneous values were recorded). Overall, we conducted 15 measurement routes (11 in Israel and 4 in Brisbane) in different nights (with good visibility and no fog or rain), covering a total of 458 km (Table 1). For each measurement point we also added the following information: whether it was located in a tunnel, the type of road it was located on (based on the ‘fclass’ attribute of the roads layer in OpenStreetMap; [23]), the municipality it was found in, whether it was an urban street/road or a highway (usually outside cities and villages, or highways cutting through a city), and whether it was a lit road or not (using the threshold of 0.1 Lux as measured by the top sensor, S1). Note that residential streets may have short sections classified as unlit either due to power failures, street lights spaced out too far from each other, obstruction of light by trees, etc.

#### 2.2.2. Space Borne Imagery

We used Sustainable Development Goals Satellite 1 (SDGSAT-1) Glimmer multispectral night-time imagery, offering a medium spatial resolution of 40 m (available from http://www.sdgsat.ac.cn/; URL accessed on 2 October 2023 [7,8]) to compare with the ground-based measurements of artificial lights and to examine the contribution of different sources of artificial lights on the signal measured by the satellite (Figure 1). We used all cloud-free SDGSAT-1 night-time images which were available for the study areas: five night-time images covering central Israel (12 February 2022, 27 March 2022, 19 May 2022, 27 February 2023, 27 April 2023) and three images covering Brisbane (9 April 2022, 17 June 2022, 18 July 2022). All images were cloud free within the study areas. Given that the ground measurements were taken within several months of the satellite acquisitions, it can be reasonably assumed that in most areas the lighting infrastructure did not change significantly between the two. We calibrated the DN values of SDGSAT-1 to apparent radiance using the sensor’s calibration coefficients of gain and bias, as provided by [8] and in the metadata files accompanying the satellite images. We summed the three calibrated spectral bands to obtain the total amount of apparent radiance measured by the SDGSAT-1. Given the inherent temporal variability in night-time lights, for each LAN3V2 ground measurement point we calculated the average apparent radiance as measured by the various SDGSAT-1 images available. We also calculated the ratio between the red and green bands of the SDGSAT-1, which we compared with that calculated from the LAN3V2.

#### 2.2.3. Supporting Datasets

We classified measurement locations into their respective road classes, based on the ‘fclass’ attribute of the roads layer in OpenStreetMap, as downloaded from https://www.geofabrik.de/ [23] (URL accessed on 22 May 2023). The ‘fclass’ attribute of the roads in OpenStreetMap contains many values. Some were not relevant for us (e.g., Footways and Track) and some we merged (e.g., Primary and Primary Link). In most of our analysis, we focused on the following six classes that represent the majority of sealed roads which are usually lit (in descending order of their hierarchy): motorway, trunk, primary, secondary, tertiary, and residential. We further classified the measurements into commercial and non-commercial locations based on Google Maps Points of Interest (POIs). To this end, we used https://outscraper.com/ (URL accessed on 22 May 2023) to extract the locations of the following categories of POIs from Google Maps, which represent commercial activities: bank, bar, café, club, fuel station, hotel, municipal, parking, pub, public transportation system, restaurant, shopping mall, sport, store, and supermarket. We extracted these locations for Brisbane (Australia) and for the following cities in Israel where our field measurements were performed: Bat Yam, Givatayim, Holon, Jerusalem, Lod, Mevaseret Zion, Petah Tikva, Ramat Gan, Ramla, Rosh Ha’Ayin, Tel Aviv. Overall, 7599 commercial POIs were extracted. These were then rasterized to the same spatial resolution of the SDGSAT-1 images (40 m). Each LAN3V2 measurement was classified as belonging to a commercial road section if it was located within a pixel where a Google Maps commercial POI was found.

### 2.3. Spatial and Statistical Analysis

The various analyses we conducted are summarized schematically in a flow chart (Figure 1). We ran an ANOVA test on log-transformed values of lux measurements and on radiance as measured by the SDGSAT-1 to find whether illuminance values varied between different road types and between urban roads and streets in Israel and in Australia (excluding tunnels, unlit road sections, and highways from this analysis). We log-transformed the lux measurements as they are commonly used to log-transform data that ranges over several orders of magnitude for better visualization and modelling [24,25]. We computed Spearman’s rank correlation coefficients to examine univariate correlations between variables, and we ran stepwise regression models to test the relationships between explanatory variables (e.g., directional lux measurements) and response variables (e.g., SDGSAT-1 night-time apparent radiance). In order to test for differences in the variability in night-time light brightness and color between urban and non-urban road sections, we used Levene’s non-parametric test. We conducted the test separately for road sections with LED street lights (*n* = 18,509 measurements) and for road sections of HPS (high pressure sodium) street lights (*n* = 12,650 measurements), which we distinguished based on our field measurements (only for Israel, because in Brisbane we did not conduct measurements in non-urban road sections); this test only included road sections for which we were able to determine the lighting technology.

## 3. Results

### 3.1. Comparisons between Road Classes and between Countries

**Figure 5:**
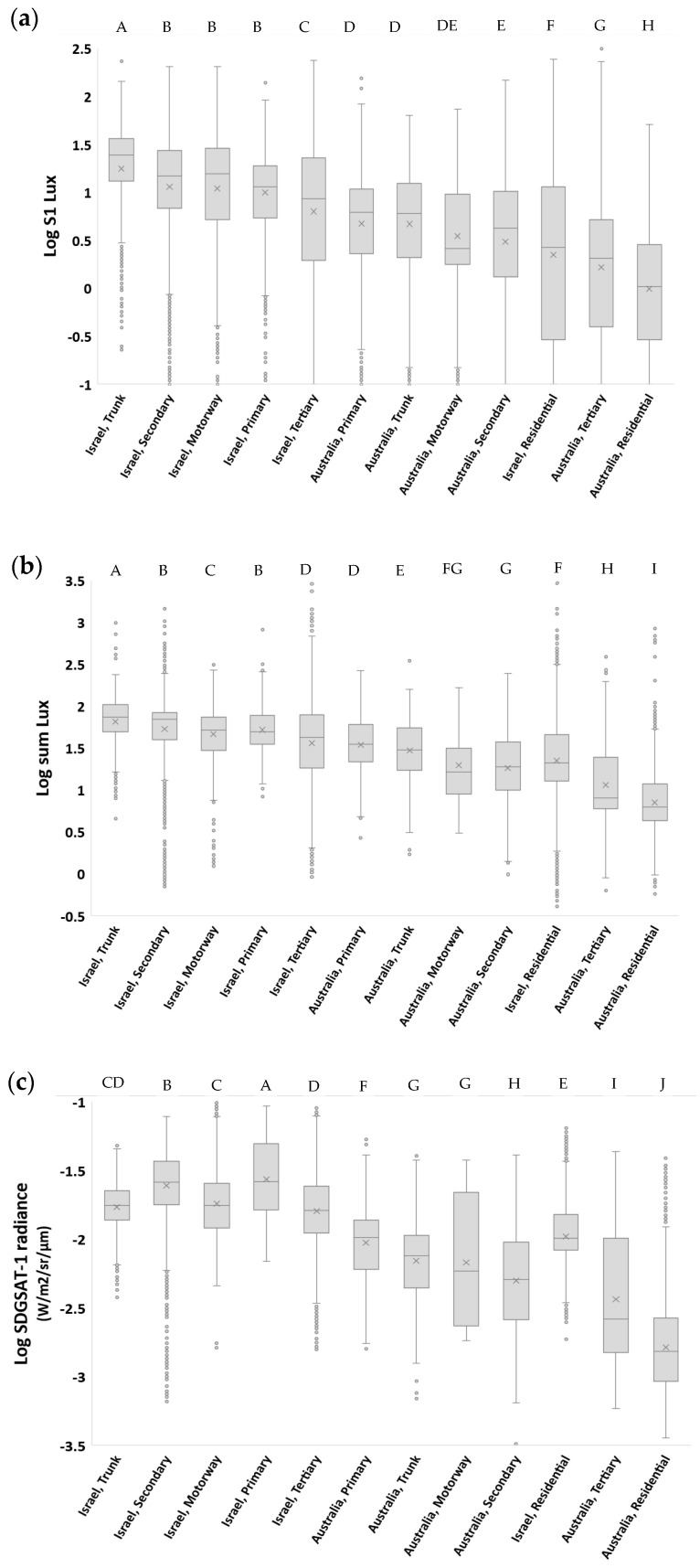
Boxplot of the lux values of the top sensor (S1) (**a**), of the sum lux values of all five sensors (**b**) of the LAN3V2, and of the radiance as measured by the SDGSAT-1 (**c**) as a function of the road type and country. This analysis includes only lit road sections located in urban areas (excluding highways and excluding tunnels) (*n* = 47,299). Categories not sharing the same letter were significantly different in their night-time brightness, based on an ANOVA and Tukey’s test. In all figures, the order of the road classes was kept the same as in Figure 4a to ease comparison. In the boxplots, the ‘x’ mark represents the average of a data set, and the ‘o’ mark represents outliers of a data set.

Ground-based illuminance measurements and satellite-based apparent radiance measurements varied between road types and between countries (Figure 5). For the three metrics we examined (S1 lux, sum lux, and SDGSAT-1 apparent radiance), major roads had higher night-time brightness values than residential streets, and when comparing the two countries for each of the road types, a certain road class always had higher night-time brightness values in cities and localities in Israel when compared with Brisbane, Australia (Figure 5). For example, residential streets in cities and localities in Israel were 4.5 times more brightly lit (as measured by the S1 lux) than in Brisbane, Australia and emitted 4.6 times the radiance as measured by the SDGSAT-1 satellite.

### 3.2. Correlations between Ground-Based and Spaceborne Brightness Measurements

We examined the correspondence between the ground-based directional lux measurements with the night-time radiance as measured by the SDGSAT-1 for all roads, roads in Brisbane and for the roads in Israel (all, highways, and urban). The summed lux values presented higher correlations with satellite measurements than any of the directions on their own (except for the subset of highways in Israel) (Table 2). Running a stepwise linear regression, the model using all directional sensors had a much higher explanatory power than a model based on the first variable which entered the model (S1 lux in all cases); overall, 62.5% of the variability in night-time radiance, as measured from above, was explained by directional lux measurements (Table 2).

### 3.3. Comparisons between Ground Based Directional Measurements

The correlations between light brightness (lux values) and color (R/G ratio) as measured in the different directions by the LAN3V2 were low, with a median Spearman’s correlation coefficient of 0.25 (Table 3 and Table 4). Overall, the correlations between the different directions were higher for lux measurements (median correlation of 0.302) than for the R/G ratio (median correlation of 0.221) (Mann–Whitney U-test *p* < 0.001). The highest correlations were found for lux measurement in urban roads lit by HPS street lights (median correlation of 0.497) (Table 3).

**Figure 6:**
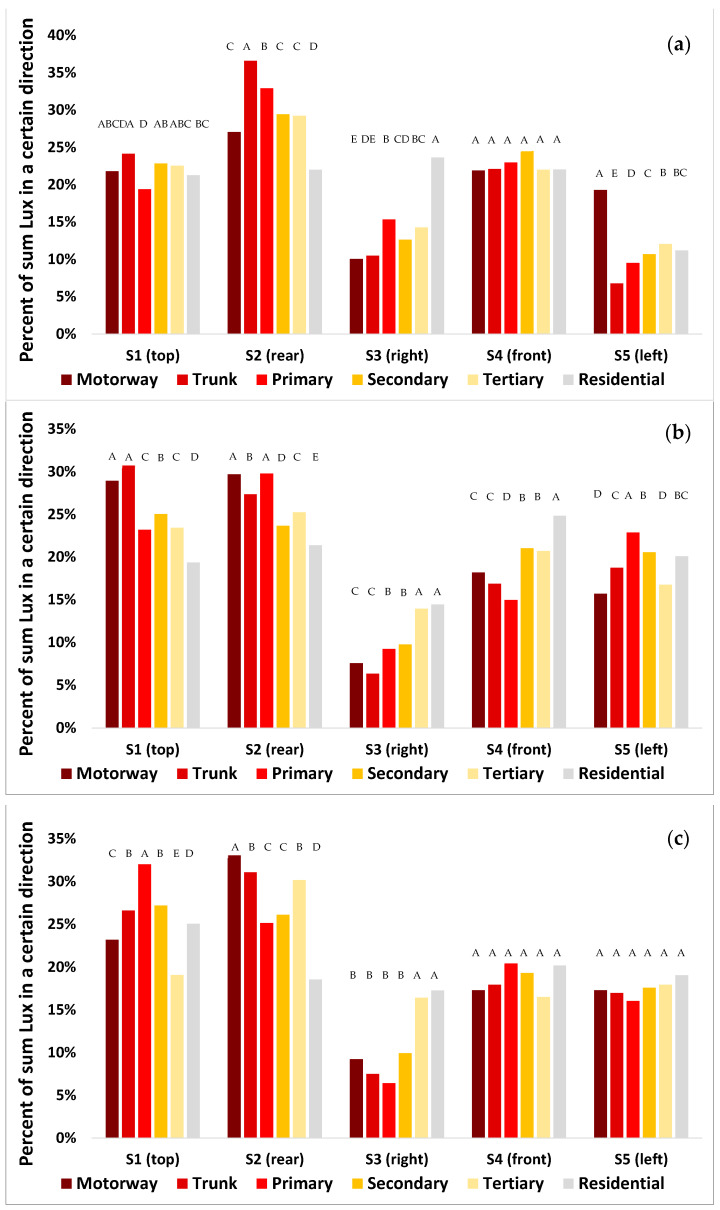
The contribution of the lux as measured by each of the directional sensors to the overall lux (sum of the five directions) as a function of the type of road in which the measurements were taken. In each sub-figure, the percentages of each of the road types sum to 100%. The top (**a**) shows urban roads in Brisbane, Australia; the middle (**b**) shows urban roads in Israel; and the bottom (**c**) shows highways outside of urban areas in Israel. This analysis includes only lit road sections and excludes tunnels. Categories not sharing the same letter were significantly different in their night-time brightness based on an ANOVA and Tukey’s test.

The amount of light that was measured in the different directions by the LAN3V2 varied between different road classes, between countries (Israel vs. Australia), and between urban roads and highways (Figure 6). Summarizing the average percentages shown in Figure 6 based on the directional sensors, the median percent contribution was highest for S2 (rear; 28.3%), followed by S1 (top; 23.3%), S4 (front; 20.6%), S5 (left; 17.1%), and S3 (right; 10.3%). In almost all cases, more than 20% of the measured light was measured both in the rear direction and upwards, whereas in most cases, less than 15% of the incoming light was measured on the right side (Figure 5). In residential streets, the contribution of light measured from the rear (S2) was always less than in major roads, whereas the contribution of light measured from the right (S3) was almost always greater in residential streets than in major roads (Figure 5).

### 3.4. Comparisons between Commercial and Non-Commercial Locations

**Figure 7:**
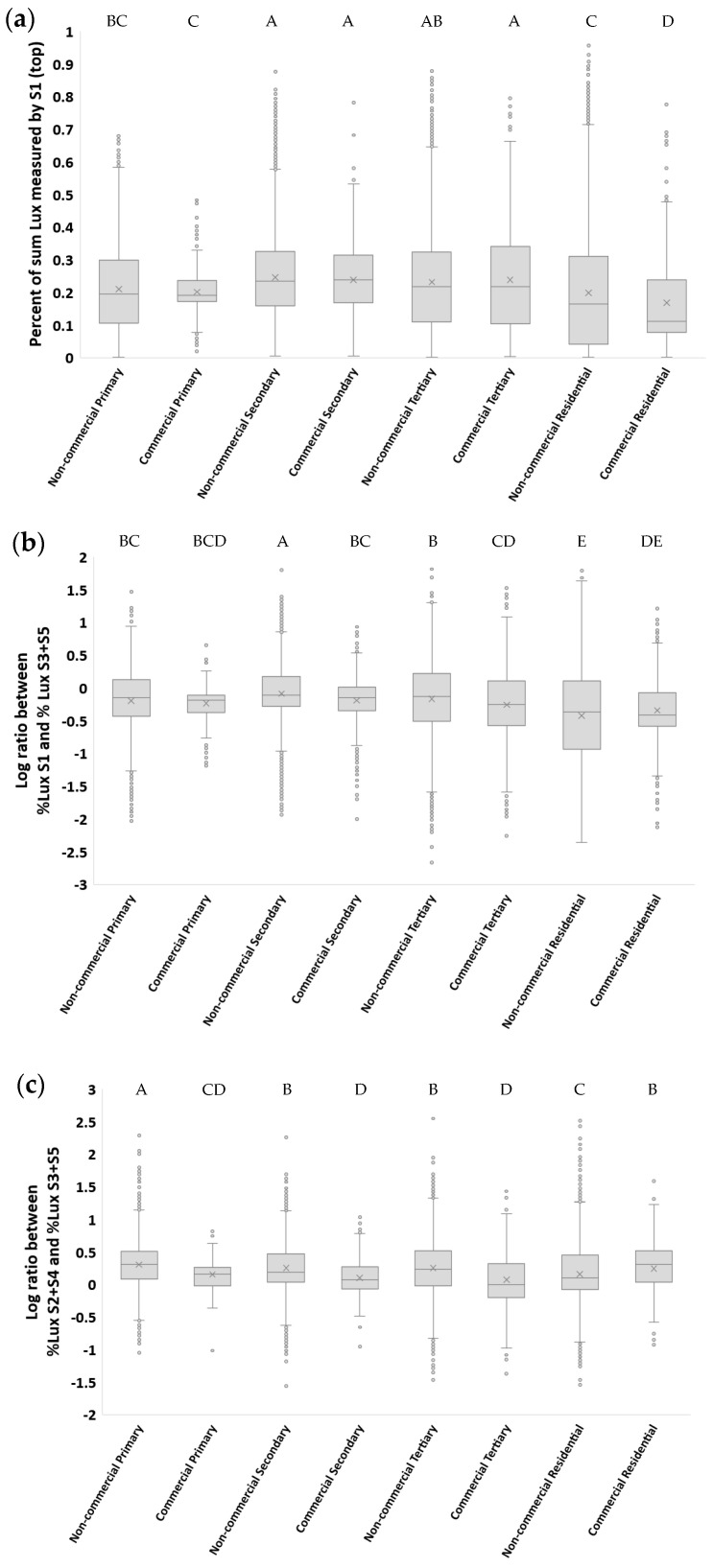
Boxplot of the percent contribution of artificial light as measured from different directions, comparing commercial and non-commercial sites in primary, secondary, tertiary, and residential streets (Israel and Brisbane, Australia). (**a**) Percent of sum lux measured by S1 [top]; (**b**) log of the ratio between the percent of sum lux measured by S1 and the percent of sum lux measured by S3 and S5 (left and right); (**c**) log of the ratio between the percent of sum lux measured by S2 and S4 (rear and front) and the percent of sum lux measured by S3 and S5 (left and right). This analysis includes only lit road sections located in urban areas (excluding highways and excluding tunnels), in four road classes (n = 43,795). Categories not sharing the same letter were significantly different in their night-time brightness based on an ANOVA and Tukey’s test. Measurements were classified as commercial or non-commercial based on Google Maps (see Methods). In the boxplots, the ‘x’ mark represents the average of a data set, and the ‘o’ mark represents outliers of a data set.

Comparing measurements in commercial and non-commercial locations, the contribution of S1 (top) to sum lux was only different in residential streets, being higher in non-commercial sites than in commercial sites (19.9% and 16.8%, respectively; Figure 7a). The ratio of S1/(S3 + S5) was higher in non-commercial locations than in commercial locations in both secondary and tertiary roads (Figure 7b). The ratio of (S2 + S4)/(S3 + S5) was higher in non-commercial locations than in commercial locations for all major road classes (primary, secondary, and tertiary); however, the opposite was found for residential streets (Figure 7c).

### 3.5. Comparisons between Urban and Non-Urban Road Sections

**Figure 8:**
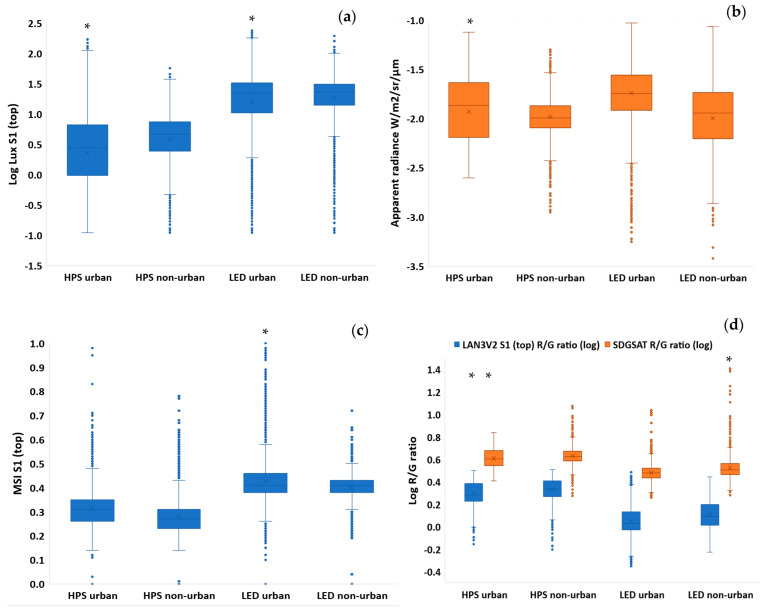
Box-plots showing the variability in brightness and color of night lights, comparing urban and non-urban roads in Israel for HPS and LED street lighting. The comparisons are between the measurements in urban and in non-urban road sections. If the variances were found to be significantly different between the urban and non-urban road sections (using Levene’s non-parametric test), an asterisk is presented over the road type with the greater variability: (**a**) lux values as measured by the LAN3V2 S1 (top) sensor; (**b**) apparent radiance as measured by the SDGSAT-1; (**c**) MSI values as measured by the LAN3V2 S1 (top) sensor; (**d**) R/G ratio values as measured by both the LAN3V2 S1 (top) sensor and the SDGSAT-1. In the boxplots, the ‘x’ mark represents the average of a data set, and the ‘o’ mark represents outliers of a data set.

**Figure 9:**
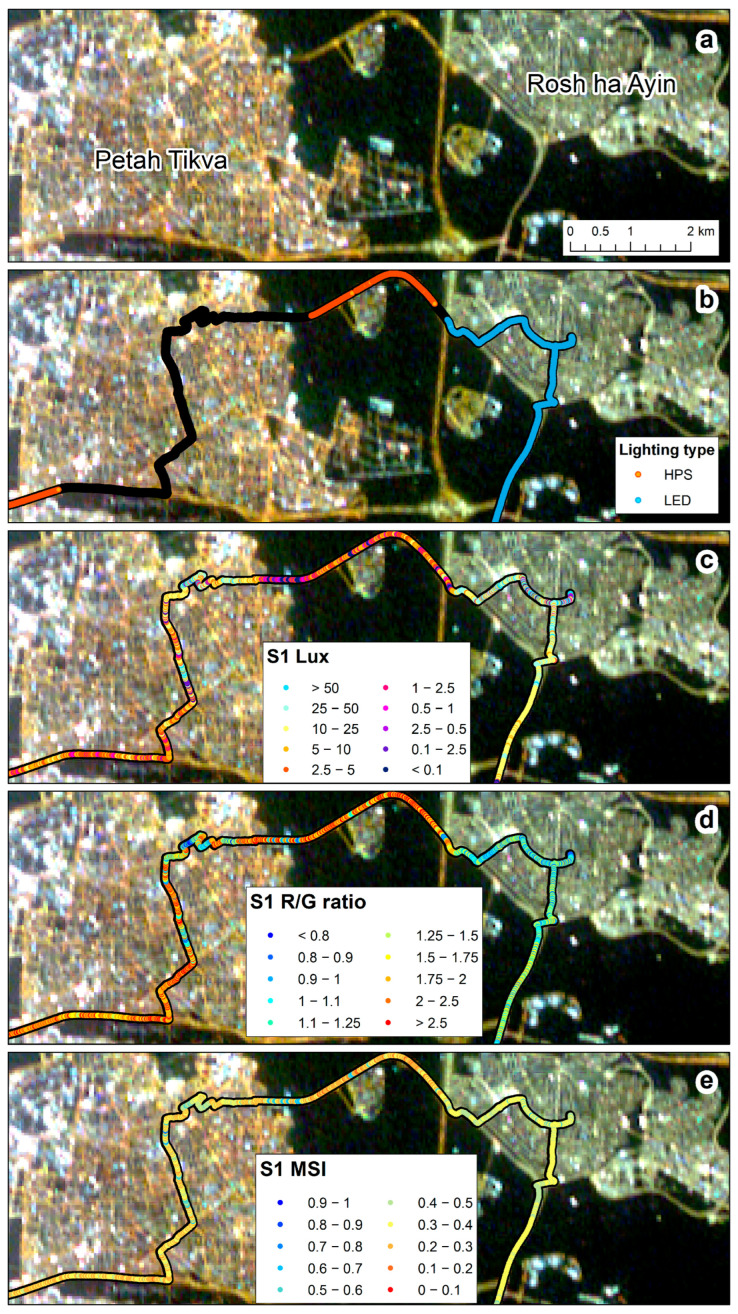
The variability of night-time brightness and color between urban and non-urban roads and between a city which is mostly lit by LED street lighting (Rosh ha Ayin) and a city where HPS street lights are still partly used (Petah Tikva): (**a**) SDGSAT-1 image of 27 February 2023; (**b**) street lighting types (measurement locations in black indicate sections where we have not identified the street light type); (**c**) LAN3V2 S1 (upwards) measurements of lux; (**d**) LAN3V2 S1 (upwards) R/G ratio; (**e**) LAN3V2 S1 (upwards) melatonin suppression index (MSI).

**Figure 10:**
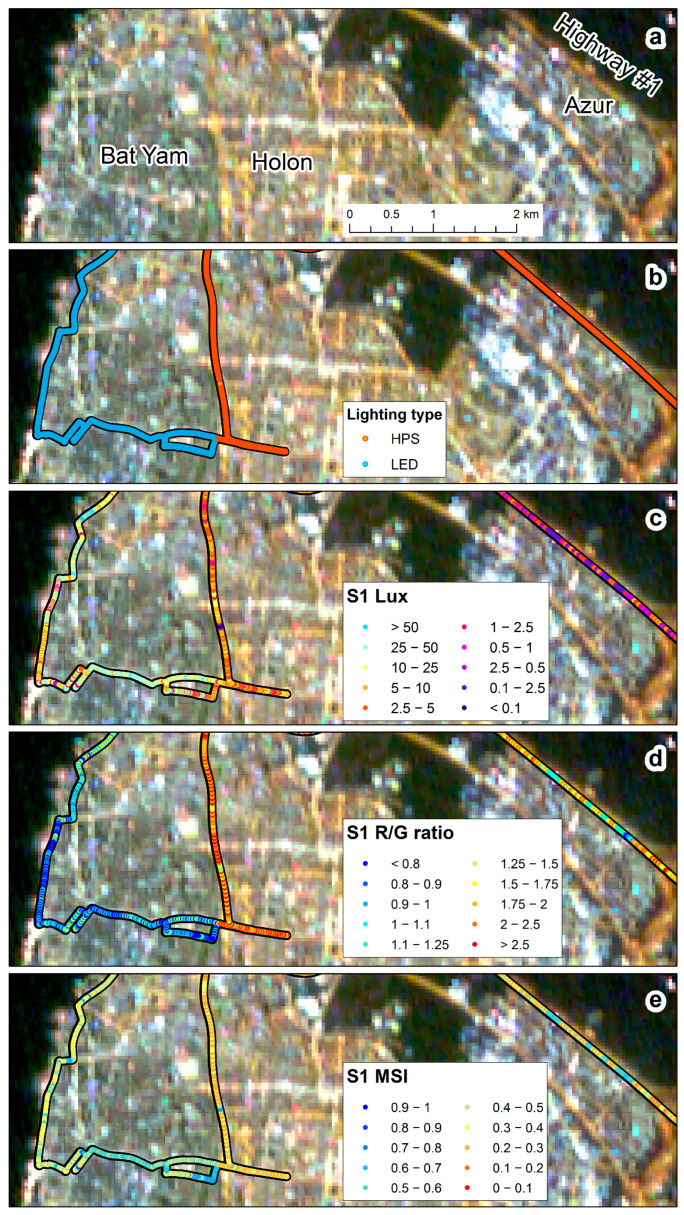
The variability of night-time brightness and color between urban and non-urban roads and between a city which is mostly lit by LED street lighting (Bat Yam) and a city where HPS street lights are still partly used (Holon): (**a**) SDGSAT-1 image of 27 February 2023; (**b**) street light types; (**c**) LAN3V2 S1 (upwards) measurements of lux; (**d**) LAN3V2 S1 (upwards) R/G ratio; (**e**) LAN3V2 S1 (upwards) melatonin suppression index (MSI).

Comparing measurements of night-time brightness in urban and non-urban road sections in Israel, we found that the variability was higher in urban road sections than in non-urban road sections for both HPS and LED street lighting (based on the LAN3V2 measurements; Figure 8a, Figure 9, and Figure 10), whereas using the SDGSAT-1 apparent radiance, variability was greater only for urban road sections lit by HPS (Figure 8b, Figure 9, and Figure 10). Using the LAN3V2, it was evident that road sections lit by LED street lights were brighter than road sections lit by HPS street lights (Figure 8a, Figure 9, and Figure 10), while using the SDGSAT-1, there were no significant differences in the apparent brightness of these two lighting technologies. As for the color, based on the LAN3V2 derived MSI, variability was greater for urban road sections of LED street-lights than for non-urban road sections (Figure 8c, Figure 9, and Figure 10). Note that MSI values were significantly higher for the LED-lit road sections than for the HPS-lit road sections (Figure 8c, Figure 9, and Figure 10). As for the R/G ratio, variability was greater for HPS-lit urban road sections than for non-urban road sections both based on the field measurements and on the SDGSAT-1 measurements (Figure 8d, Figure 9, and Figure 10). For LED-lit road sections, the variability was greater in the non-urban road sections than in the urban road sections, only when calculated based on SDGSAT-1 measurements (Figure 8d, Figure 9, and Figure 10). Note that based on both the LAN3V2 and the SDGSAT-1 measurements, R/G ratio values were higher for HPS-lit road sections than for the LED-lit road sections (Figure 8d, Figure 9, and Figure 10).

## 4. Discussion

The LAN3V2 photometer is a relatively new ground-based instrument which offers a unique capability of measuring night-time brightness in three spectral bands and in five directions, thus allowing to map artificial lights and their colors and related light pollution indices over a wide area [22]. Working in a rural area, Aubé and Houle [26] demonstrated that using its multidirectional measurements, it was possible to map street light inventories in two small villages. In this paper, we examined the variability in night-time lights (both brightness and color) as measured by the LAN3V2 to better understand the factors affecting it and its correspondence with spaceborne measurements of night lights, as measured by the SDGSAT-1 [7]—the first multispectral spaceborne sensor offering acquisition of night-time lights at a medium spatial resolution as suggested in 2007 [27].

While ground measurements of night lights in each of the five measured directions were correlated with space-borne measurements of apparent radiance (Rs > 0.5; Table 2), a multivariate model using all directional sensors had a stronger explanatory power than a model using only the S1 sensor (which was directed upwards, towards street lights). The need to include light measurements in all directions to explain night-time brightness as measured from space confirms that while street lights are an important source of artificial lighting, they are one of many light sources [18]. The multivariate model was better in explaining spaceborne measurements of night lights over highways (R^2^ = 0.725) than for urban roads (R^2^ = 0.556); this may be explained by the greater variability in light sources within the city, the large contribution of horizontal lights in urban areas [28] that may not reach satellites, by the vertical structure of cities leading to obstruction of some light sources by buildings, and by the emission of light both upwards to light iconic buildings and horizontally from windows of high-rises [29]. The need to include light measurements from different directions to better explain spaceborne measurements was also confirmed by the relatively low correlations between light as measured in the different directions (Table 3). In the study of Katz and Levin [17], where SQM measurements were taken in three directions (upwards, downwards, and horizontal), it was found that horizontal light measurements were indeed more informative than upwards measurements to explain night-time brightness as measured by the EROS-B sensor. Both upwards and horizontal measurements of night-time brightness are characterized by high variability because they are exposed to point sources, whereas downwards measurements are less variable [30] and better correlated with spaceborne measurements [17]. However, analyzing night-time measurements in all directions is needed to better characterize the light pollution to which we (humans) and other organisms are exposed.

Previous studies have documented that the night-time brightness of major roads is higher than that of residential streets [31]. We found similar results here, both for roads in Israel and for roads in Brisbane, based on ground measurements as well as based on the SDGSAT-1 imagery (Figure 5). The horizontal lighting sources also varied between major and residential roads, with the percent contribution of lights from the back (representing cars driving behind) being much lower on residential streets than on major roads (Figure 6), presumably because of lower traffic in residential streets. In most cases, the two directions from which most of ground-based measured light was coming was either from the back or from above (representing street lights). The differences in the direction of incoming light sources presents a new approach to estimate land use in urban areas. Previous studies often focused on differences between land use classes based on the apparent radiance as measured from night-time aerial photos (e.g., [32]) or from space (e.g., [6,7,13,31]), or based on night-time spectral ratio indices [7,13]. In a recent paper [33], both night-time brightness and angular effects as measured by the VIIRS/DNB were used for identifying central business districts. Here, we examined the potential of ground based directional measurements to differentiate between land uses. Of the three ratios we examined, the best differentiation between commercial road sections and non-commercial road sections was based on the ratio of (S2 + S4)/(S3 + S5); on major roads (primary, secondary, and tertiary), commercial road sections were characterized by a reduction in the contribution of incoming light from cars (either from the front or the rear) relative to an increase in the contribution of light coming from the sides (left and right), presumably from lit shops and associated commercial lighting used to attract attention [34,35].

The higher correlations between directional measurements found for lux measurements in urban road sections lit by HPS lighting than for road sections lit by LED lighting (Table 3) may result from the better directionality of LED street lighting and the greater light spillage from HPS street lighting [36]. Based on the field measurements, the variability in night-time brightness was greater in urban road sections than in non-urban road sections (regardless of the lighting technology), which confirms again the greater variability in lighting sources in urban areas [37]. This variability in lighting sources, which become mixed in the pixels of night-time images, makes it difficult to quantify the intensity and even more so the color of night-time lights from space, especially in urban areas.

Ground measurements of night-time brightness as part of remote sensing studies have often used sky quality meters (SQM; [17,28,30]) or lux meters [28,38], such as the LAN3V2 [22,26], for calibration and validation purposes. While both approaches are valid, one of the main differences between them relates to the sensitivity threshold of illuminance meters vs. that of the SQM. The SQM is sensitive to very low levels of light and can measure natural dark skies (starlit and overcast, >21 magSQM/arcesc2, equivalent to less than 0.01 lux; [19]), whereas the lowest thresholds for illuminance meters and for spaceborne night-time sensors are usually much higher (e.g., 0.015 lux for the LAN3V2; [22]). As a result of this threshold difference, in very dark conditions, illuminance meters and night-time spaceborne sensors may not record meaningful data, whereas the SQM will still be able to quantify night-time brightness levels and variability (compare with [17]). As a result, in areas which appear to be dark on spaceborne imagery, SQM readings will always show variability, regardless of their viewing direction. Therefore, illuminance meters may show better correlations with night-time spaceborne sensors than SQMs. However, instruments such as SQMs are needed to measure dark sky conditions, as required for the establishment of dark sky parks [39].

Comparing the night-time brightness as measured both on the ground and from space between Israel and Australia confirmed previous studies (which used the VIIRS/DNB sensor) which found that cities in Israel were much brighter than cities in Australia [10] and that a much larger percentage of the population lives under very high night-time light intensities with no dark adaptation for human eyes (>3000 mcd/m^2^) in Israel (61% of the population) compared to Australia (13.1% of the population) [40]. While the brightness levels of cities’ nightscapes are very different between Israel and Australia, both are high-income-economy countries belonging to the OECD (The Organisation for Economic Co-operation and Development). In lower-income-economy countries, street lights are often not centrally planned and maintained uniformly [41], and therefore it may be expected that there will be lower correlations between socio-economic properties and night lights in such countries (see the comparison between Arab and Jewish localities in Israel and the West Bank; [11]).

**Figure 11:**
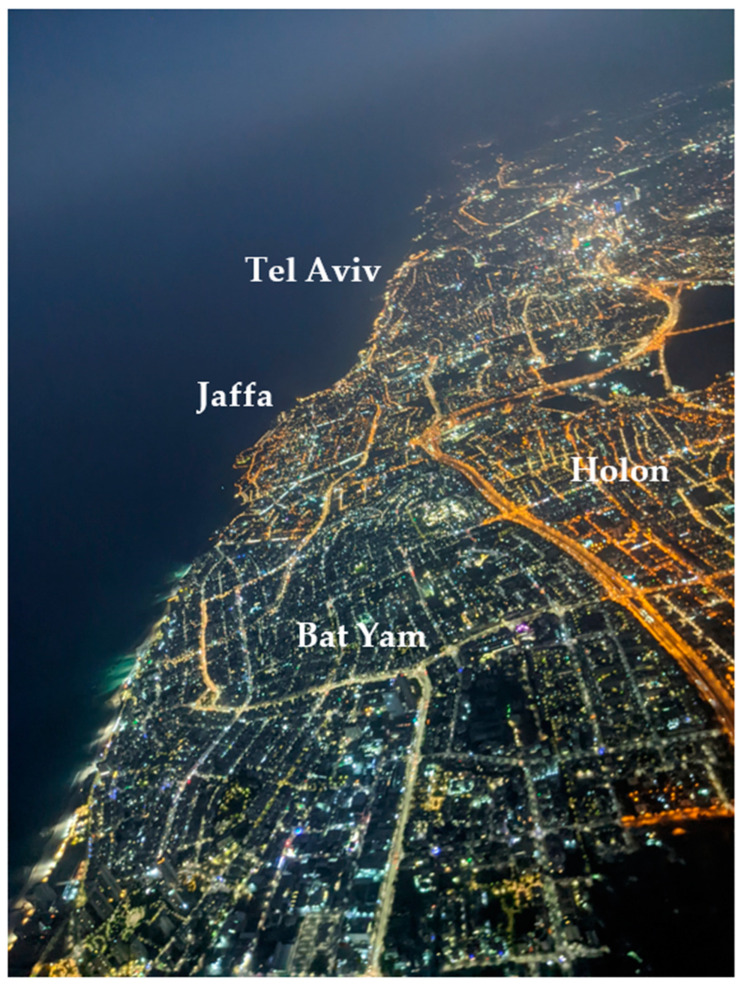
Aerial night-time photo acquired on 28 October 2022, contrasting the night-time lights of Bat Yam (mostly LED street lights) and Holon (mostly HPS street lights). Note the spatial variability in night-time light brightness and color. Photo taken by Shahar Har’el.

With the transition of street lights and other lighting sources around the world to LED technology, one of the challenges is to identify changes in the lighting technology and to quantify changes in the overall brightness and color of the nightscape [4,42,43]. Brightness-wise, our ground measurements confirmed that upward illuminance (from street lights) was greater in road sections lit by LED technology than in road sections lit by HPS technology (Figure 8a, Figure 9 and Figure 10; as expected by [4]). However, these differences were less evident when comparing the apparent radiance as measured by the SDGSAT-1 (Figure 8b, Figure 9 and Figure 10). The lower performance of SDGSAT-1 in distinguishing between the brightness of HPS and LED street lighting may be explained as follows: The ground measurements with the LAN3V2 S1 (top) sensor mostly represent light emitted from street lights above the car. The night-time measurements of the SDGSAT-1 are based on a larger footprint (pixels of 40 × 40 m) and thus incorporate light which is both emitted and reflected not only from street lights but also from other light sources (e.g., from buildings, commercial areas, etc.). In addition, the SDGSAT-1 imagery is not atmospherically corrected, and hence the measurements are affected by atmospheric scattering. As for the change in the color of the light, both the MSI (measured by the LAN3V2) and the R/G ratio (measured both on the ground and from space) indicated that road sections in Israel which have transitioned from HPS to LED lighting had a lower component of red light (Figure 8c,d, Figure 9 and Figure 10). However, the variability in the R/G ratio was greater in urban road sections than in non-urban road sections (for road sections lit by HPS) based on both the LAN3V2 and the SDGSAT-1. The greater variability in lighting in urban road sections than in non-urban road sections indicates that identifying changes in lighting technologies within urban areas from space, especially at a medium spatial resolution (as that of the SDGSAT-1), is challenging, given that within each pixel there is a mix of lighting sources, complex vertical structures and obstructions, and a greater variability in surfaces of different materials, color, and textures from which artificial light is reflected (compare Figure 10 and Figure 11).

## 5. Conclusions

With the ongoing transition of the world’s nightscape to LED lights, the recent availability of both spaceborne multispectral sensors (e.g., SDGSAT-1) and ground-based multispectral and multidirectional sensors (e.g., LAN3V2) is key, as they enable us to map in greater detail both the brightness and the color of the night. These richer data sources allow us to better characterize and understand the variability in night-time lights, which varies between urban and non-urban roads as well as between commercial and non-commercial road sections.

## Figures and Tables

**Table 1:** Descriptive statistics of the measurement routes included in this study.

			Percent of All Measurements			
Route Name	Date	Km	Unlit (Lux S1 < 0.1)	OSM Road Class		
				Motorway, Trunk, Primary	Secondary, Tertiary	Unclassified, Residential, Service
Israel1	11 January 2022	3.3	15.3%	0.0%	11.6%	88.4%
Israel2	12 January 2022	3.3	0.0%	0.0%	88.5%	11.5%
Israel3	24 November 2022	19.8	2.4%	18.7%	57.9%	23.4%
Israel4	3 December 2022	14.1	0.4%	40.1%	18.0%	41.9%
Israel5	13 December 2022	127.0	6.7%	67.1%	16.6%	16.3%
Israel6	18 December 2022	9.9	56.6%	55.9%	32.5%	11.6%
Israel7	28 December 2022	33.2	25.1%	26.3%	59.2%	14.5%
Israel8	2 January 2023	13.7	0.2%	56.2%	28.9%	14.9%
Israel9	18 January 2023	12.2	9.6%	21.0%	54.1%	24.9%
Israel10	20 January 2023	35.3	0.5%	18.0%	63.4%	18.6%
Australia1	21 February 2023	13.5	10.2%	0.0%	30.8%	69.2%
Australia2	21 March 2023	8.3	0.0%	0.0%	57.4%	42.6%
Israel11	31 March 2023	4.6	0.0%	0.0%	75.8%	24.2%
Israel12	14 April 2023	3.9	0.4%	0.0%	62.6%	37.4%
Australia3	18 April 2023	26.2	10.2%	32.4%	47.4%	20.2%
Australia4	23 April 2023	12.5	4.7%	37.8%	48.1%	14.1%
Israel13	28 April 2023	11.2	0.1%	63.2%	30.3%	6.5%
Israel14	12 May 2023	3.1	0.0%	0.0%	64.7%	35.3%
Israel15	15 May 2023	103.1	15.5%	51.6%	29.9%	18.6%
Total		458.2	9.3%	33.8%	40.1%	26.0%

**Table 2:** The relationships between directional lux measurements and the night-time radiance as measured by the SDGSAT-1. The top six rows show univariate Spearman’s rank correlation coefficients between the directional lux measurements and SDGSAT-1 radiance. The next two rows show the adjusted R2 of a linear regression model, in which SDGSAT-1 radiance values (log-transformed) served as the response variable, and the directional measurements of lux values (log-transformed) served as the explanatory variables. All analyses did not include tunnels.

		All Roads	Brisbane	Israel	Israel—Highways	Israel—Urban
Spearman rank correlation coefficients	S1 Lux (top)	0.591	0.505	0.581	0.535	0.560
	S2 Lux (rear)	0.608	0.629	0.601	0.519	0.614
	S3 Lux (right)	0.596	0.560	0.632	0.708	0.510
	S4 Lux (front)	0.596	0.605	0.592	0.503	0.581
	S5 Lux (left)	0.678	0.647	0.638	0.590	0.618
	Sum Lux	0.703	0.712	0.692	0.626	0.670
Linear regression Adjusted R^2^	S1 Lux	0.420	0.227	0.467	0.603	0.346
	Full model using all directional sensors	0.625	0.526	0.644	0.725	0.556
	n	82,108	14,330	67,778	27,073	40,705

**Table 3:** Spearman’s rank correlation coefficients between LAN3V2 lux measurements in the different directions for roads lit by HPS street lights and for roads lit by LED street lights. The correlations were calculated for road sections in Israel for which the street lighting was known. In each 5 × 5 correlation matrix, the correlation values above the diagonal (i.e., in the upper-right half of the matrix) refer to non-urban road sections, and the correlation values below the diagonal refer to urban road sections. All correlations were statistically significant.

	HPS					LED				
	S1	S2	S3	S4	S5	S1	S2	S3	S4	S5
S1		0.40	0.37	0.36	0.23		0.30	0.51	0.48	0.20
S2	0.61		0.32	0.22	0.28	0.30		0.39	0.24	0.16
S3	0.42	0.43		0.20	0.20	0.34	0.26		0.29	0.23
S4	0.52	0.37	0.49		0.24	0.31	0.22	0.23		0.21
S5	0.65	0.56	0.33	0.50		0.29	0.24	0.17	0.21	

**Table 4:** Spearman’s rank correlation coefficients between LAN3V2 R/G ratio measurements in the different directions for roads lit by HPS street lights and for roads lit by LED street lights. The correlations were calculated for road sections in Israel for which the street lighting was known. In each 5 × 5 correlation matrix, the correlation values above the diagonal (i.e., in the upper-right half of the matrix) refer to non-urban road sections, and the correlation values below the diagonal refer to urban road sections. All correlations were statistically significant.

	HPS					LED				
	S1	S2	S3	S4	S5	S1	S2	S3	S4	S5
S1		0.08	0.23	0.06	0.17		0.39	0.22	0.08	0.35
S2	0.16		0.36	0.13	0.16	0.30		0.36	0.08	0.21
S3	0.26	0.28		0.24	0.22	0.27	0.29		0.20	0.08
S4	0.28	0.09	0.23		0.15	0.20	0.14	0.24		0.27
S5	0.27	0.06	0.39	0.40		0.20	0.27	0.06	0.11	

## Data Availability

SDGSAT-1 imagery is available from https://data.sdgsat.ac.cn/ accessed on 2 October 2023.
